# Ultrasound targeted microbubbles for theranostic applications in liver diseases: from molecular imaging to targeted therapy

**DOI:** 10.1080/10717544.2025.2541656

**Published:** 2025-08-07

**Authors:** Chujun Zhang, Qiaoyu Zhang, Qiao Xu, Xinyi Jiang, Yao Ma, Chaoqi Liu, Chang Zhou, Rong Liu, Yun Zhao, Yun Liu

**Affiliations:** a Department of Ultrasound Imaging, The First College of Clinical Medical Science, China Three Gorges University & Yichang Central People’s Hospital, Yichang, China; b Hubei Key Laboratory of Tumor Microenvironment and Immunotherapy, China Three Gorges University, Yichang, China; c Medical College of China three Gorges University, Yichang, China

**Keywords:** Ultrasound-targeted microbubble destruction (UTMD), liver diseases, theranostics, contrast-enhanced ultrasound, targeted drug delivery, molecular imaging

## Abstract

Liver diseases, particularly chronic conditions leading to cirrhosis and hepatocellular carcinoma, represent a major global health burden with high mortality rates, necessitating innovative diagnostic and therapeutic approaches. Ultrasound-targeted microbubble destruction (UTMD) technology has emerged as a promising theranostic platform, combining enhanced contrast imaging with targeted drug/gene delivery capabilities. When activated by ultrasound, these microbubbles exhibit unique biophysical behaviors that significantly improve drug penetration, tissue perfusion, and site-specific delivery. This review comprehensively examines recent advancements in UTMD-based strategies for liver disease management, including microbubble design and imaging-targeted functionalization, and mechanisms of ultrasound-enhanced drug delivery, especially emerging theranostic applications. We further discuss the underlying biophysical principles governing microbubble-ultrasound interactions and their translational potential, providing insights for developing next-generation precision medicine approaches for hepatic disorders.

## Graphic Abstract

**Figure:**
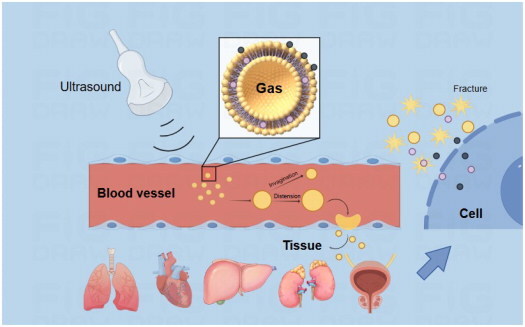

## 1. Introduction

**Figure 1.**
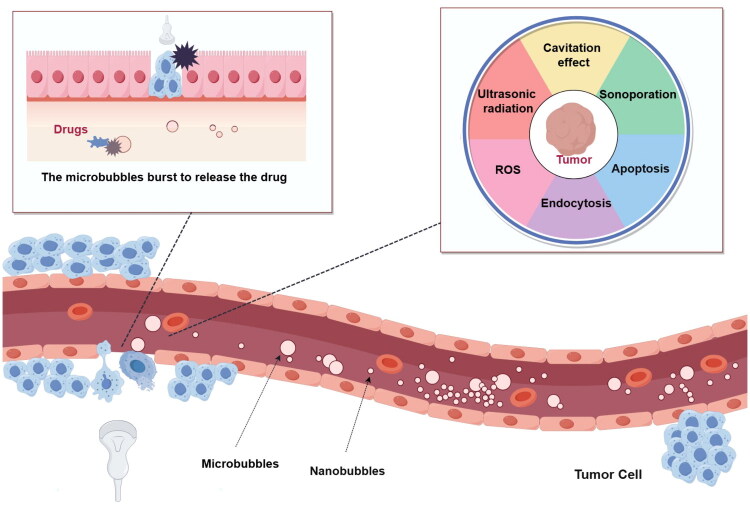
UTMD irradiated microbubbles by ultrasound, resulting in microbubble rupture. This process not only releases the drug, but also increases cell membrane permeability through the resulting microjets and shock waves. In addition, cavitation effect, acoustic pore effect and thermal effect are used to achieve the purpose of treatment. ***Drawn by the authors***.

UTMD (Figure 1) is an ultrasound therapy technique that uses low frequency ultrasound (20 kHz to 1 MHz) to stimulate the cavitation of microbubbles in vivo, resulting in vibration of vapor or gas cavities under the action of acoustic waves (Paliwal and Mitragotri 2006). The acoustic impedance of microbubbles differs significantly from that of soft tissues and has reduced attenuation of low-frequency harmonics, which can increase their contrast with surrounding tissues. Microbubbles can therefore be used as contrast agents to visualize the anatomy of organs and tissues, i.e., ultrasound contrast agents (UCAs) (Battaglia and Cervelli 2017; Quaia 2019; Rix et al. 2020). After ultrasound irradiation, microbubbles in the body produce an ultrasonic cavitation effect. Cavitation contributes to microbubbles thrombus penetration, drug delivery, local acoustic perforation, and increases cell membrane permeability. In additional, ultrasound also produces Acoustic Radiation Force (ARF), which can be used to manipulate the displacement of nanoparticles and cells in solution (Wu and Nyborg 2008; Chowdhury et al. 2020; Rix et al. 2020). Studies have shown that ARF can push ligand-labeled microbubbles to the blood vessel wall and significantly enhance the image signal. In addition, acoustic radiation force can also be used as a mechanical destructive agent to destroy the integrity of endothelial cells (Urban 2018; Sarvazyan et al. 2021). And ultrasonic microbubbles are an excellent delivery vehicle for bioactive molecules (such as drugs, acoustic sensitizer, and therapeutic genes) to organs or tissues with ultrasound, which could enhance the therapeutic efficacy of the proposed combination therapy for liver diseases (Urban 2018; Pellow et al. 2020; Sarvazyan et al. 2021).

Liver disease is a serious global public health problem, accounting for 4% of all deaths worldwide, mainly including viral hepatitis, liver fibrosis, and hepatocellular carcinoma (Wang et al. 2014; Xiao et al. 2019). Among them, hepatocellular carcinoma is the third leading cause of cancer-related deaths, while cirrhosis and liver fibrosis are also highly lethal (Parola and Pinzani 2019; Devarbhavi et al. 2023). Currently, hepatocellular carcinoma responds poorly to existing treatments, which calls for new therapies. Ultrasound-targeted microbubble disruption (UTMD) has established itself as a versatile diagnostic and therapeutic platform capable of providing real-time imaging guidance while localizing liver disease (Xing et al. 2024). For example, delivery of nanoparticles of tiny RNA in a mouse model of hepatocellular carcinoma modulates the immune microenvironment of the tumor and enhances the efficacy of combination therapy (Wischhusen et al. 2020).

## 2. Preparation techniques and application of ultrasonic microbubbles

Microbubbles (microbubbles) are a new type of drug delivery vehicle, usually consisting of an internal gas core and a surfactant shell (Fournier et al. 2023). It is spherical in shape, ranging from ten nanometers to several microns in diameter, and can exist in a relatively stable structure in a liquid. Because the microbubble particle size is small, it can pass through the biofilm barrier and easily enter the tissue (Shi et al. 2020; Lin et al. 2023). Targeted microbubbles are coupled with targeted peptides, antibodies or other ligands to their membranes. Specific peptides, antibodies or other ligands attached to the surface of microbubbles or wrapped inside microbubbles are selectively binding to highly expressed antigens, receptors and specific markers of the targeting cells, blood vessels and microenvironment in the target tissue by ultrasound irradiation, finally the targeted drug was delivered to the lesion site (Paliwal and Mitragotri 2006; Hernot and Klibanov 2008; Rix et al. 2020; Fournier et al. 2023). Due to the incomplete vascular basement membrane, increased vascular endothelial cell space (about 380–780 nm) and lack of lymphatic drainage, the enhanced permeability and retention effect (EPR) of microbubbles made it preferable passively to accumulate in the tumor site (Chowdhury et al. 2020), improve the targeting efficiency of drugs or genes, and promote the accumulation of microbubbles in the tumor to achieve the effect of targeted therapy (Hernot and Klibanov 2008; Han et al. 2024).

### 2.1. Production of ultrasonic microbubbles

#### 2.1.1. Material selection of ultrasonic microbubbles

In order to maintain certain stability and good biocompatibility of microbubbles in vivo, the carrier microbubble materials (Table 1) include lipids, polymers, lipid-polymer hybrids, proteins and inorganic nanoparticles. At present, the commonly used microbubble shell materials are composed of lipids or albumin, which are mixed and arranged into single or multi-layers. The widely used lipid materials include phospholipid mixtures such as DSC (1,2-dioleoyl-sn-glycero-3-phosphocholine) and DSPE-PEG2000 (1,2-distearoyl-sn-glycero-3-phosphoethanolamine-N-[methoxy(polyethylene glycol)-2000]. Due to the changeable shell composition of microbubbles, the different component lipid monolayers will affect the stability of microbubbles. And the interaction between the hydrophobicity of microbubbles and van der Waals force can not effectively prevent gas diffusion and accumulation (Upadhyay and Dalvi 2019), so the lipid shell of microbubbles has not only lipid flexibility but also weak immunogenicity. It can prevent microbubbles from being cleared by the reticulo-endothelial system. The structural stability of microbubbles depends on shell and gas, which is affected by shell elasticity, microbubble surface tension and viscosity of aqueous solution. Sridhar et al. found that in suspensions the stability of microbubbles can be increased by thickening aqueous solution by adding harder shell materials (such as polymers) and glycerol (Fournier et al. 2023).

**Table 1.** Materials for microbubbles.

Material type	Specific materials	Purpose/Features
Shell Materials	Phospholipid (e.g.DSC, DSPE-PEG2000)	Formation of single or multi-layer films; High biocompatibility
	Proteins (e.g.Albumin)	Good stability; Drug delivery
	Polymers (e.g.PLGA, PLA, PCL)	High mechanical strength; Biodegradability
	Inorganic Nanoparticles (e.g.SiO_2_, Metal nanoparticles)	High mechanical strength; Good stability;
Gas Core Material	Perfluorocarbons (e.g. C_3_F_8_, C_4_F_10_)	High stability; Low solubility
	Atmosphere	Low cost; Poor stability
	Helium	Moderate stability;
Stabilizer	Glycerol	Improved stability
	Sorbitol	Prevents microbubble aggregation

#### 2.1.2. Preparation techniques of ultrasonic microbubbles

Influenced by a variety of preparation methods, microbubbles often have different physical and chemical properties. At present most of the microbubbles studied are liposomes with gas cores. The main synthesis method is to dissolve a certain proportion of the lipid mixture into an organic solvent, use a rotary evaporation system to obtain a dry membrane, and then hydrate the dry lipid membrane to help the lipid re-suspension. Ultrasound is used to shake the solution containing lipids evenly to make microbubble suspension, which has the advantages of low cost, high synthesis rate and fast synthesis speed (Fournier et al. 2023). There is also a microbubble preparation method based on inkjet printing technology, which can produce polymer nano-bubbles with small particle size. First, inkjet particles are prepared, add the core components of organic solvents that evaporate slowly, and then the core components are removed by freeze-drying. Because the microbubble structure does not contain a waterborne core, the microbubbles can only encapsulate hydrophobic drug molecules. On this basis, the polymer shell is filled with perfluoro pentane, which is liquid at room temperature and becomes gaseous at body temperature. The perfluoro pentane is extravasated to the tumor through intravenous administration, and the drug is released from the degradable polymer shell under ultrasonic treatment.

Shaking technology is also a viable method in commercial ultrasonic microbubbles, Definity^®^ microbubbles are synthesized by shaking a lipid solution in a sealed vial. In addition, it has been documented that electrohydrodynamic atomization generates lipid microbubbles by applying a constant electric field to a lipid solution in contact with air (Stride et al. 2020).

During the process of preparing microbubbles, it is inevitable to generate nanobubbles. These bubbles, when disturbed, will aggregate to form micrometer-sized bubbles. Therefore, it is very difficult to control the preparation of macroscopic microbubbles or nanobubbles. In recent years, the combination of different preparation methods has gradually gained attention. This can effectively increase the bubble production and control the bubble size, making the application scenarios more diverse, reducing the production cost, and lowering the operating energy consumption. For example, Deng et al. (2023) prepared a super-stable three-layer structure protein microbubble through an ethanol-water exchange strategy. This strategy can regulate the size of the bubble core by adjusting the proportion of ethanol. In addition, methods such as thin film hydration, aerosol method, and electrochemical method have also become increasingly mature (Omata et al. 2020).

### 2.2. Application of ultrasonic microbubbles

#### 2.2.1. Microbubbles for diagnosis

In 1968, it was found that small echo air bags were formed after injection of normal saline, which could significantly improve the contrast of ultrasound imaging (Kooiman et al. 2014). This was the first time that microbubbles had been observed in the body. In the past, some scholars have studied the distribution of albumin microbubbles and red blood cells in the blood vessels of golden hamsters. Because the distribution velocity and frequency of microbubbles in the lumen of golden hamsters were similar to those of red blood cells, microbubbles were used as an intravascular tracer in echocardiography (Rix et al. 2020). At present, contrast-enhanced ultrasound (CEUS) has been used in clinic for 25 years, and has become a routine auxiliary method of gray-scale ultrasound and color Doppler ultrasound. Due to the great difference of high sound impedance between gas and blood or soft tissue, microbubbles become a contrast agent for ultrasonic imaging, that is used to increase the backscattering of signals, which is much larger than that of blood and tissue organs. The nanovesicle ability of extravasating into the tissue space through blood vessels has been much studied in cancer therapeutics, and enhanced penetration and retention (EPR) effects associated with tumor endothelial leakage with a few hundred nanometer opening have been found. Therefore, microbubbles with ideal size can be used for specific imaging of tumors, which is safe and easy to implement. Microbubble-assisted ultrasound imaging has been widely used in clinic because it can provide real-time quantitative information of biological tissue morphology and blood perfusion, and observe different type of disease or abnormal tissue microcirculation perfusion.

**Figure 2.**
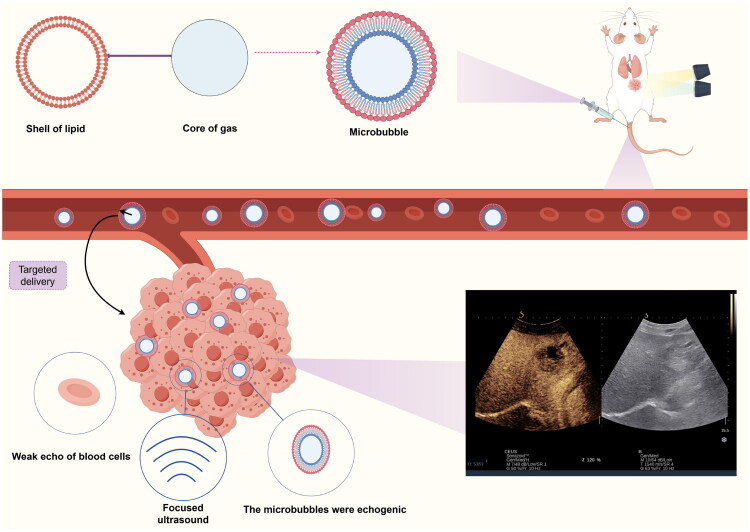
The echogenicity of microbubbles is enhanced after ultrasound irradiation, which can significantly improve the resolution, sensitivity and specificity of ultrasound diagnosis. After intravenous injection, a high-density area can be transiently formed, which is convenient for disease diagnosis. ***Drawn by the authors***.

The use of contrast-enhanced microbubbles (Figure 2) is called ultrasound contrast agent (UCAs), which is usually composed of tiny bubbles about 2–6 μ m in diameter and has a short half-life in the blood, usually only a few minutes. Unlike CT iodine contrast agent and MRI gadolinium contrast agent, which cannot pass through the blood vessel wall, the ultrasound contrast agent is very small and can easily pass through the micro-vessels and enter the interstitial space (Fournier et al. 2023). The ultrasound contrast agent consists of a gaseous core and a surfactant shell, which can stabilize the structure of microbubbles, and slow down their dissolution in the blood. When UCAs enters the tissue, when it is irradiated by ultrasound in a specific area, these tiny bubbles will appear nonlinear oscillation. Because their expansion is greater than their contraction, the echo produced by the harmonic oscillation will be the fundamental frequency and the multiple of this frequency, which makes the acoustic impedance difference between the gas core and the surrounding tissue more significant. Increasing the signal-to-noise ratio of tissue and microbubbles is helpful to distinguish the echo of microbubbles from the background tissue. Secondly, compared with the background tissue, harmonic oscillation leads to the echo amplification of microbubbles by several orders of magnitude, which makes the signal from microbubbles easier to be detected and received by the instrument, which is not only used to delineate the boundary of the body cavity and increase the vascular Doppler signal. It can also improve the sensitivity of microcirculation, and can be used to evaluate various diseases of heart disease, radiology, and oncology.

##### 2.2.1.1. Commercial diagnostic microbubbles

Microbubbles are ultrasound contrast agents (UCAs) composed of gas-filled cores stabilized by shells (e.g., phospholipids, albumin). Key FDA/EMA-approved microbubbles include: SonoVue^®^ (Bracco Imaging), Definity^®^ (Lantheus Medical Imaging), Sonazoid^®^ (GE Healthcare), Optison^®^ (GE Healthcare). Bracco’s Sonovue^®^ vial (Bracco, Geneva, Switzerland), a phospholipid shell microbubble that wraps the core of sulfur hexafluoride (SF_6_) gas and can be used for heart, vascular and liver imaging. The Definity^®^ vial (Lantheus Medical Imaging, Billerica, Mass), which has a phospholipid shell, but its gas core is octafluoropropane (C_3_F_8_), is also approved for ultrasonic imaging of the heart, abdomen, and pelvis. There are also two types of microbubbles, Sonazoid^®^ vial (GE Healthcare AS, Oslo, Norway) and Optison^®^ vial (GEHealthcareAS, Oslo, Norway), which have a lipid shell with perfluoro butane (C_4_F_10_) gas core and an albumin shell with perfluoro propane (C_3_F_8_) gas core, respectively. Sonazoid^®^ vial is mainly used for abdominal imaging and Optison^®^ vial for cardiac imaging. At present, SonoVue and Definity are suitable for the diagnosis of focal liver lesions. The specific method is used to inject microbubbles into the elbow vein, and rinse with 5–10 ml normal saline after the injection to remove the residual contrast agent in the venous luma. A low power contrast agent with a mechanical index of 0.0 8 ∼ 0.12 is adopted, and the timing starts from the normal saline irrigation. Dual screen image contrast mode and B-ultrasound image mode were used to help guide and determine the location of nodules. If the location of the lesion is difficult to show in the arterial phase due to the small size of the lesion, or the patient is unable to cooperate with holding the breath, or there is evidence of nodules of unknown nature in the portal vein phase, a second microbubble injection may be performed to help diagnose the nature of the nodules. Almost all UCAs are pure blood drugs and are not metabolized by the kidney, so contrast-enhanced ultrasound is the preferred diagnostic method for patients with renal insufficiency. In addition, since ultrasound imaging is dynamic and real-time, CEUS is also recommended for patients with inconclusive CT or MRI results.

Some diagnostic applications of microbubbles in Clinical Trials are summerized in Table 2. For instance, trials like NCT00788697 and NCT00829413 evaluated SonoVue^®^-enhanced US for characterizing focal liver lesions, demonstrating its diagnostic utility with a low incidence of serious adverse events (1.48% and 1.18%, respectively). Similarly, NCT02321527 employed Perflutren Protein-Type A Microspheres for sentinel lymph node imaging in breast cancer, reporting no adverse events. Other studies, such as NCT05328167 and NCT06639828, are exploring microbubbles (e.g., Sonazoid^™^) for liver and pediatric lesion diagnosis, highlighting their role in improving lesion detection and monitoring treatment responses.

**Table 2.** Clinical trials and safety of UTMD.

National clinical trial (NCT) number	Tumor/condition	Purpose (diagnosis/treatment)	Contrast agent/device	Conclusion/status	Serious adverse event
NCT03458975	Colorectal Cancer, Hepatic Metastases	Targeted Delivery of Chemotherapy with Ultrasound and Microbubbles (Treatment)	SonoVue^®^	Results not specified	/
NCT00788697	Liver Neoplasms	*SonoVue^®^-Enhanced vs. Unenhanced US for Focal Liver Lesion Characterization (Diagnosis)*	SonoVue^®^	With Resultes	1.48% (5/337, eg. Metabolic acidosis; Dehydration; Preemptive syncope; Hemothorax, etc.)
NCT00829413	Liver Neoplasms	*SonoVue^®^-Enhanced vs. Unenhanced US for Focal Liver Lesion Characterization (Diagnosis)*	SonoVue^®^	With Resultes	1.18% (4/340, eg. Dehydration; Hemorrhage, etc.)
NCT05328167	Intrahepatic Cholangiocarcinoma	Contrast-Enhanced US for Predicting Response to Radioembolization (Diagnosis/Treatment Monitoring)	Perflutren Protein-Type A Microspheres	Ongoing	/
NCT03477019	Breast Neoplasms, Colorectal Neoplasms	Ultrasound-Enhanced Chemotherapy Delivery for Liver Metastases (Treatment)	SonoVue^®^	Results not specified	/
NCT02321527	Breast Cancer	*CEUS Sentinel Lymph Node Imaging with Guided Biopsy (Diagnosis)*	Perflutren Protein-Type A Microspheres	With Resultes	0.00% (0/21)
NCT02967458	Prostatic Neoplasm	Subharmonic US Imaging for High-Grade Prostate Cancer Detection (Diagnosis)	Perflutren Protein-Type A Microspheres	With Resultes	/
NCT03335566	Liver Lesions	Efficacy/Safety of Sonazoid^™^ vs. SonoVue^®^ for Pre-/Post-Contrast US Imaging (Diagnosis)	Sonazoid^™^ SonoVue^®^	With Resultes	/
NCT06639828	Focal Liver Lesions (Pediatric)	Sonazoid Safety/Efficacy for CEUS Liver Imaging (Diagnosis)	Sonazoid^™^	Ongoing	/
NCT04440358	Recurrent Glioblastoma	Exablate BBB Disruption + Carboplatin (Treatment)	Focused ultrasound	Ongoing	/
NCT06496971	Recurrent Glioblastoma, Brain Tumors	Bevacizumab ± Microbubble-mediated FUS (NaviFUS System) (Treatment)	Microbubble	Ongoing	/
NCT04417088	Recurrent Glioblastoma	Exablate BBB Disruption + Carboplatin Monotherapy (Treatment)	Microbubble Resonators	Ongoing	/
NCT05317858	NSCLC Brain Metastases	BBB Opening with Exablate FUS + Standard Therapy (Treatment)	Sulfur hexafluoride microbubbles	Ongoing	/
NCT05630209	Pediatric DIPG	BBB Disruption with Exablate FUS + Doxorubicin (Treatment)	Microbubble Resonators	Ongoing	/
NCT05615623	Pediatric DIPG	BBB Disruption with Exablate FUS + Doxorubicin (Treatment)	Microbubble Resonators	Ongoing	/
NCT06329570	Recurrent Glioblastoma	Bevacizumab + NaviFUS System (Treatment)	Lumason^®^	Not yet recruiting	/

All the above information can be accessed through the ClinicalTrials.gov website. https://clinicaltrials.gov/expertsearch?term=ultrasound%20and%20targeted%20microbubbles&page=3.

In liver disease, CEUS is primarily used to diagnose local intrahepatic masses, such as unexpectedly found benign lesions of the liver (hemangiomas and focal nodular hyperplasia) on grayscale ultrasound. The European Society of Medical Ultrasound and the World Federation of Medical Biology strongly recommend the use of contrast-enhanced ultrasound to diagnose hepatocyte nodules in patients with liver fibrosis. It should also routinely be used to identify focal liver lesions that are uncertain or suspected to be malignant after routine ultrasound and every intrahepatic lesion detected during ultrasound monitoring in patients with a history of liver disease (chronic liver disease, cirrhosis, or malignant tumor) (Quaia 2019). Contrast-enhanced ultrasound was recently approved by the U.S. Food and Drug Administration for the diagnosis of some liver diseases in adult and pediatric patients. The Enhanced Ultrasound Liver Image Reporting and Data System (LIRADS) can be used to provide a structured report of HCC diagnosis to better assist in the diagnosis and differentiation of HCC. Compared with CT and MRI, CE ultrasound has considerable overall accuracy in the characterization of focal liver lesions, higher sensitivity in the identification of malignant tumors, and higher specificity in the exclusion of malignant tumors.

CEUS are used to assist in the detection and evaluation of intrahepatic lesions, which are distributed in specific phases as follows (Table 3). Because of the double blood supply of hepatic portal vein and hepatic artery, the imaging pattern of different stages has unique significance and information. Arterial staging (AP) can observe the degree and pattern of arterial blood supply in focal hepatic lesions (FLL) and show the arterial morphology of focal arterial stage tumors. In the portal venous phase (PVP), UCAs reaches the hepatic parenchyma through the portal vein system, maximizing the diffuse enhancement of normal hepatic parenchyma. The late phase shows the process by which ultrasound contrast agent is cleared from the body’s circulation, while the post- vascular phase is only observed in contrast agent Sonazid angiography and represents the process by which ultrasound contrast agent is absorbed by phagocytes such as Kupffer cells. Hepatic ultrasound contrast agent is to compare the contrast agent with the adjacent hepatic parenchyma, enhance the vascular structure and specific stage of the lesion, and provide very important information for the diagnosis and evaluation of the nature of auxiliary lesions.

**Table 3.** Vascular phase in contrast-enhanced ultrasound of the liver (visualization post-injection time).

Phase	Start (s)	End (s)
Arterial	10–20	30–45
Portal venous	30–45	120
Late	>120	Bubble disappearance (approximately 4–8 min)
Post-vascular	>8min	Approximately 30 min

##### 2.2.1.2. Self-made diagnostic microbubbles

With the passage of time, the vigorous development of molecular biology makes ultrasonic molecular imaging become a research hotspot. The study found that microbubbles carrying biomarkers can be widely used in the diagnosis and evaluation of diseases such as cancer, vascular injury, or inflammation. Therefore, researchers are committed to combining diagnostic ultrasound contrast agents with cellular molecular targeting markers to generate specific effects at the target site through designed biomarkers, and locally to concentrate microbubbles, thus achieving local imaging of the target area (Langeveld et al. 2021). For example, microbubbles are coated with platelet membranes, and then these hybrid microbubbles are endowed with various adhesion receptors (such as integrin αIIbβ3) for the early diagnosis of acute kidney injury(SAKI) by selective adhesion to endothelial cells with SAKI caused by sepsis (Yang et al. 2021). Sentinel lymph nodes may be labeled with targeted microbubbles carrying the breast cancer specific biomarker B7-H3 to assist the diagnosis and evaluation of breast cancer (Hu et al. 2022), or microbubbles carrying thymic cell antigen 1(Thy1) can be used specifically to diagnose pancreatic tumors (Bam et al. 2020). In addition, specific adhesion proteins produced by vascular diseases such as thromicrobubblesosis, atherosclerosis or endothelial inflammation can also be used as targets of microbubbles to assist in disease diagnosis (Nunn et al. 2004; Köse et al. 2020). This self-made microbubble carrying markers makes the microbubbles have a great application prospect as a contrast agent for molecular imaging.

#### 2.2.2. Therapeutic microbubbles

Some Therapeutic Applications of Microbubbles in Clinical Trials are summerized in Table 2. In therapeutic settings, microbubbles are leveraged to enhance drug delivery or disrupt biological barriers. Trials like NCT03458975 and NCT03477019 investigated SonoVue^®^-assisted ultrasound for targeted chemotherapy delivery in colorectal cancer and liver metastases. Additionally, several ongoing trials (e.g., NCT04440358, NCT05630209) focus on microbubble-mediated blood-brain barrier (BBB) disruption (using Exablate FUS or NaviFUS systems) to improve drug penetration in glioblastoma and pediatric brain tumors (e.g., DIPG). These studies aim to combine microbubbles with agents like carboplatin or doxorubicin, offering promising strategies for treating aggressive cancers.

**Figure 3.**
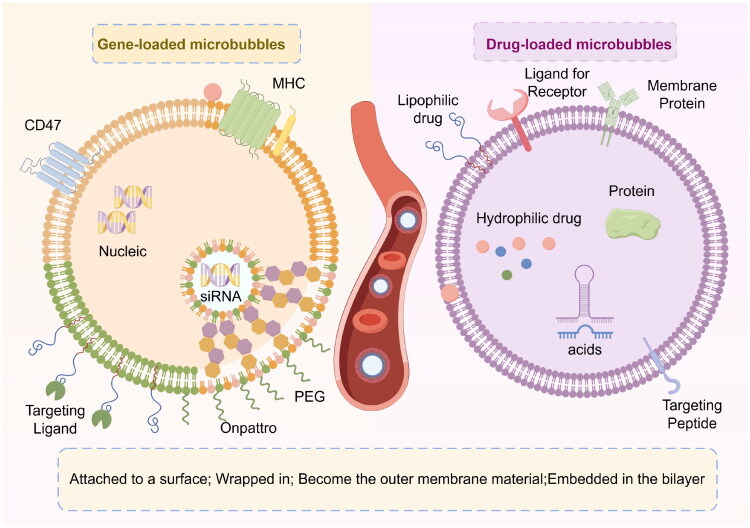
Microbubbles have powerful drug delivery functions. Microbubbles can carry drugs or genes in the following ways: (a) gene/drug is attached to the surface of microbubbles; (b) gene/drug was directly used as the shell component of microbubbles. (c) genes/drugs are embedded between the inner and outer membranes of bimolecular microbubbles; (d) gene/drug encapsulated inside the microbubble. ***Drawn by the authors***.

UTMD can cause a series of changes in vascular endothelial cells by using the cavitation effect caused by ultrasound irradiation, such as enhancing the permeability of endothelial cells, increasing the level of intracellular calcium, regulating gene expression, stimulating the activity of nitric oxide synthase and so on. These effects can promote drug penetration, increase blood perfusion, assist drug delivery, and induce tumor cell death. In addition to drugs, many genes (including DNA, siRNA, miRNA), peptides, and antibodies can be combined with targeted microbubbles for gene therapy. Although viruses have been regarded as the most effective gene carrier selection in clinical practice in previous studies, their application has been limited due to severe immune inflammation and cancer, insufficient protection against gene degradation, and insufficient improvement in effective accumulation efficiency of targeted damaged genes. Therefore, UTMD is a promising and fruitful gene delivery method that creates instantaneous pores in cell membranes and blood vessels via ultrasound (US) or ultrasound accompanied by microbubbles, thereby increasing tissue penetration and uptake of intracellular drugs and genes (Tran et al. 2019). It provides a relatively safe carrier system for gene transmission (Figure 3).

##### 2.2.2.1. Gas components involved in the therapy of microbubbles

As one of the components of microbubbles, the gas core of microbubbles usually has echo characteristics, stability, and even therapeutic properties. The diagnostic microbubbles are usually selected by inert gases with stable properties and structures, the most common being sulfur hexafluoride or perfluoro propane, which have both stable chemical structure and hydrophobic properties that can affect the half-life of microbubbles in vivo. However, in some special microbubbles, gas can not only be used to stabilize the microbubble structure, but also participate in the treatment of diseases as a therapeutic agent (Jägers et al. 2021; Fournier et al. 2023).

***① Oxygen (O_2_)*** As the tumor microenvironment is anoxic and acidic, some studies have encapsulated oxygen in microbubbles to inhibit tumor growth. In this study, oxygen-carrying lipid microbubbles were directly injected into the tumor, which significantly improved the therapeutic effect of radiotherapy. It was also found that with the increase of tumor size, the tumor became more responsive to the treatment of oxygen-carrying lipid microbubbles (Fix et al. 2015; Song et al. 2020). However, because many types of tumors are too far away from the body surface directly to inject microbubbles into the tumor, some researchers have intravenously injected microbubbles to deliver oxygen to the tumor. In a rat breast cancer model, increased radiosensitivity can be observed by injecting oxygen-carrying microbubbles into the tail vein (Delaney et al. 2019). Compared with radiotherapy alone, this method of inhibiting tumor hypoxia environment could significantly delay tumor growth. Another study also found that UTMD combined with oxygen-carrying microbubbles could increase the oxygenation rate of tumor and significantly reduce the expression of hypoxia-inducible factor-1 α and its related genes. It prevents angiogenesis and epithelial-mesenchymal transition (EMT), and inhibits tumor metastasis (Xu et al. 2022). In addition, to improve the stability of oxygen-carrying microbubbles, Peng et al mixed SF6 into the gas core, which increased the half-life of oxygen-carrying microbubbles by 3 times. It suggests that Oxygen delivery therapy has a prospect as a supplementary therapy for cancer therapy (Peng et al. 2021).
***② Nitric oxide (NO)*** Nitric oxide (NO) plays a regulatory role in angiogenesis. It is mainly used for intracellular signal transduction, angiogenesis, and vasodilation. Nitric oxide, as an effective radiosensitizer, can directly react with free radical DNA to repair damage, reduce radiation resistance caused by hypoxia, and normalize abnormal tumor blood vessels by increasing blood perfusion and oxygen supply (Bellary et al. 2023). However, due to the unstable chemical structure of nitric oxide, it is difficult to reach specific areas in the body to play a therapeutic role, limiting its application in disease treatment. A study designed a nitric oxide hydrogel reservoir, which can slowly release NO within tumors, promote vascular normalization, reverse tumor hypoxia, while increase cell sensitivity to radiotherapy, and induce tumor cell apoptosis (Yang et al. 2022). In addition, nitric oxide can stimulate endothelium and treat deep venous thrombosis by playing the role of platelet and inflammatory cell aggregation. Nitric oxide coated microbubbles have important application prospects in the treatment of deep vein thrombosis (Radi 2018; Carlström 2021).
***③ Other gases*** In addition, other gases can also be used as microbubble-encapsulated therapeutic agents in the treatment of tumors. Carbon monoxide (CO) can increase mitochondrial respiration, lead to overproduction of reactive oxygen species (ROS) and promote apoptosis. CO can increase mitochondrial respiration, lead to excessive production of ROS and promote apoptosis. CO also is a transmitter in cellular signal transduction, which can play a role in the central nervous system. Intracellular production of CO can up-regulate cyclic guanosine phosphate to protect the nerve. However, excessive concentrations of CO can lead to hypoxia and damage to brain cells, so neuroprotective therapy by encapsulating microbubbles of CO is still being studied (Adach et al. 2020; Kashfi 2022). Although inert gases such as perfluorocarbons and perfluoro propane have no direct therapeutic effect, they can be used as intermediate transporters of oxygen to help transport oxygen, and their inert properties also make their solubility low in blood and tissues, which can provide stability for microbubbles and maintain the normal structure of microbubbles (Krafft and Riess 2021; Hanieh et al. 2022).

##### 2.2.2.2. Microbubbles of drug and gene delivery

In the treatment of many diseases, the transmission and delivery of many drugs and genes (DNA, miRNA, siRNA, etc.) in vivo is limited due to the existence of biological barriers in vivo, drug toxicity, insufficient accumulation of local drug concentration and biodegradability, etc. Microbubbles are an excellent carrier in which drug molecules are incorporated, either by attaching the drug to the surface of the bubble via covalent or non-covalent bonds, or by incorporating the drug or gene into the nanoparticle and then binding to the bubble. At present, the main drug delivery strategies of drugs and genes on microbubble are as follows (Xiao et al. 2019): (a) genes bind to the surface of cationic lipid microbubbles by non-covalent binding; (b) based on the multi-layer structure of lipid microbubbles coated with genes and multiple L-lysine layers, polymer microbubbles with a hollow core and surrounded by a thick polymer shell; (c) hydrophobic drugs are loaded in the shell of polymer microbubbles; (d) hydrophilic drugs are loaded in the internal voids of polymer microbubbles; (e) the internal structure of polymer microbubbles that the aqueous phase is dispersed through the polymer matrix and distributed on multiple cavities of the microbubble structure during freeze-drying; (f) liposomes or nanoparticles are attached to the surface of microbubbles by biotin-avidin-biotin bridge.

Under normal circumstances, it is difficult for gene and drug carrier particles to penetrate through the endothelial cell layer from the blood to the organs and tissues, but studies have found that UTMD helps the microbubbles loaded with drugs or genes to promote drug penetration through the micro-fluid. The destruction of microbubbles helps to penetrate the vascular endothelial barrier in the target tissue. In addition, minor injury caused by UTMD can promote the aggregation of proinflammatory cells, platelets and bone marrow-derived stem cells in tissue, and induce angiogenesis and astrogenesis (Shi et al. 2020). Even the blood-brain barrier that can block most drugs can be reversibly ‘softened’ by ultrasound-mediated microbubble damage to improve the ability of drugs to enter the central nervous system (Yan et al. 2021). Compared with other methods, ultrasound-mediated microbubble destruction technique has little injury effect on blood vessels, and is noninvasive, safe and effective techniques.

Although ultrasound itself can increase the permeability of cell membrane, the combined application of ultrasound and microbubbles makes the effect more significant; when microbubbles are destroyed by ultrasound, microbubbles are located near the cell, and cavitation can increase the permeability of cell membrane; Secondly, the reactive oxygen species produced by ultrasound changed the permeability of the cell membrane without affecting cell survival. The thermal effect caused by the absorption and dissipation of ultrasonic energy can not only increase the local instantaneous temperature, but also affect the fluidity of cell membrane phospholipid bilayer, and then affect the cell permeability. In addition, endocytosis and phagocytosis may be involved in the absorption of microbubbles, while active transport mechanisms such as the fusion of lipid microbubbles and phospholipid cell membranes may be involved. Recent studies have also found that nano-sized microbubbles have prolonged acoustic vascular pharmacokinetics, can passively infiltrate tumor intact, actively enhance the transport of intact nano-bubbles and shell materials, and increase their space bioavailability deep into the outer space of blood vessels (Pellow et al. 2020). Acute vascular effects have also been observed, which can be used for extended imaging and therapeutic applications outside the vascular system (Zenych et al. 2020).

In addition, the use of microbubbles is not limited to tumors, and the cavitation characteristics of ultrasound microbubbles can improve the delivery efficiency of genes in gene therapy, and can be used in the treatment of various systemic diseases such as the central nervous system and digestive system (Huang et al. 2017; Kancheva et al. 2023). Nano-sized bubbles have the advantage of smaller volume during exosmosis and tissue infiltration, and their potential in tissue gene therapy has been proved by different groups (Hernot and Klibanov 2008). Kida et al improve the transmission of genetic material by optimizing the size of microbubbles and the feasibility of gene transfection in mice using hand-held ultrasound scanners (Kida et al. 2022).

Based on the above reasons, encapsulating drugs or genes in microbubbles and triggering local release, deposition, and enhancement in the target tissue by ultrasound can help to reduce the incidence of adverse events and improve the therapeutic effect. As the delivery system, microbubbles have good biocompatibility and delivery efficiency, and have great potential in oncology and vascular applications (Table 4).

**Table 4.** Drug/gene-carrying microbubbles.

Drug/gene	Microbubbles			Anti-tumor mechanisms	Tumor models	Reference
	Shell	Core	Diameter (nm)			
CD133; sPD-1gene	Lipids	C_3_F_8_	929 ± 23	T-lymphocyte activation; CD8 + T cell infiltration	Mice/cervical cancer	Liu et al. (2023)
CXCL10; IL-2; αPD-L1	Lipids	C_3_F_8_	1178	Enhances CD8+ T cell infiltration and prevents their exhaustion	Mice/Glioblastoma	Dong et al. (2024)
Gemcitabine; Anti-PD-L1 antibodies	polymers	/	160–180	Activation of CD8+ T cells; Upregulation of PD-L1 expression	Mice /Breast, melanoma Cancer	Shi et al. (2023)
Apatinib	Lipids	SF_6_	/	Apatinib combined with UTMD showed anti-angiogenesis and growth inhibition	Mice/ Breast Cancer	Hong et al. (2021)
Indocyanine Green; Paclitaxel	Lipids	C_3_F_8_	469.5 ± 32.87	NBs promoted the release of Paclitaxel. NBs promoted the apoptosis of cancer cells. NBs enables multimodal imaging	Mice/Prostate Cance	Lan et al. (2025)
Doxorubicin	Lipids PLGA	NH_4_HCO_3_	800	Doxorubicin induced immunogenic tumor death. The Lipid/PLGA MBs multi-void structure enhanced cavitation effect and drug encapsulation efficiency	Mice/ Breast Cancer	Chen et al. (2019)
Paclitaxel; Doxorubicin; Rose Bengal	Lipids	O_2_	/	O2MB increased ROS content. Paclitaxel and Doxorubicin inhibited the proliferation of tumor cells.	Mice/ Breast Cancer	Logan et al. (2019)

### 2.3. Microbubbles for theranostics

Because of the diverse functions of microbubbles, some researchers have begun to integrate diagnostic microbubbles with therapeutic agents for theranostic applications. In 2023, Tang employed the ultrasound-induced perfusion effect to improve tumor blood perfusion and enhance the delivery of doxorubicin. By activating microbubbles with ultrasound, a cavitation effect was induced within the tumor tissues of rats, thereby significantly improving perfusion efficiency and facilitating increased transport of doxorubicin (Tang et al. 2023). The peak intensity of CEUS can also be increased, and similar studies also demonstrated in the reference (Jamburidze et al. 2019). Because microbubbles are usually intended either as macroscopic imaging methods or for gene or drug delivery, their precise localization of acoustical active components in and out of blood vessels is not apparent. Pellow demonstrated effective exosmosis of acoustically active nanobubbles, whose exosmosis components not only consist of bubble fragments and payloads, but also found the exosmosis of destructive cavitation of microbubbles through dynamic evaluation. They also described the enhancement effect of ultrasound stimulation on microbubbles extravasation, the contrast enhancement of tumors was 1.5 times that of baseline, and the contrast enhancement of ultrasound stimulation could reach 5 times that of baseline, which also had certain significance in imaging (Pellow et al. 2018). More research groups combined the passive and active targeting of tumors, and utilized the acoustic response characteristics of microbubbles to assist tumor diagnosis and treatment. Chen et al. covalently coupled anti-PD-L1 antibodies to nanoparticles, and utilized the highly expressed PD-L1 in tumors to reduce tumor immune escape, while conducting molecular imaging for tumor immunotherapy (Chen et al. 2022). In additional, the size of microbubbles and nanoparticles with different shells and properties will also affect imaging (Navarro-Becerra et al. 2022), so the influence of their diameters is constantly being explored. In the future, by better controlling the acoustic effect of nanoparticles, nanoparticles can be applied to differential imaging or therapy at the same time.

## 3. The application of UTMD in liver diseases

### 3.1. UTMD is used in the diagnosis and treatment of liver fibrosis

#### 3.1.1. Imaging of liver fibrosis

Liver fibrosis is a dynamic process influenced by multiple factors, including the interaction of persistent chronic liver injury, chronic inflammation, and progressive fibrosis, and this extensive chronic liver injury can induce extracellular matrix (ECM) protein accumulation and eventually lead to cirrhosis (Koyama and Brenner 2017; Fournier et al. 2023). Therefore, it is essential early to detection and accurate staging of liver fibrosis. Due to its convenience and noninvasive nature, ultrasound may be one of the most suitable tools for liver fibrosis monitoring. Ultrasound elastography, which is widely used at present, is a promising technique for staging liver fibrosis, but in the early stage of fibrosis, the liver is affected by inflammation and necrosis in addition to fibrosis. Ultrasound elastography mainly reflects the changes of liver structure through liver hardness, but these early fibrosis changes only show abnormalities at the molecular and cellular levels, which cannot be detected by elastic ultrasound. Therefore, an imaging method that can monitor and quantify the occurrence and progression of liver fibrosis at the molecular and cellular levels is needed to compensate for the shortcomings of ultrasound elastography.

In the studies, contrast-enhanced ultrasound (CEUS) with perflubutane was used to diagnose liver fibrosis in rats (Liu et al. 2013), and basic ultrasound and liver and spleen enhanced ultrasound images were obtained. Compared with conventional ultrasound, the sensitivity of CEUS was increased from 63% to 84%, and the accuracy was increased from 71% to 88%. Previous studies have shown that angiogenesis is closely related to liver fibrosis (Koyama and Brenner 2017). One study specifically demonstrated molecular and cellular changes of liver fibrosis through ultrasonic molecular imaging (USMI) to monitor angiogenesis at the cellular and molecular levels (Qiu et al. 2021). In some studies, Anti-VEGFR2 monoclonal modified lipid microbubbles (microbubbles) have been synthesized as imaging probes for early hepatic fibrosis. Vascular endothelial growth factor receptor 2(VEGFR2), as a classical mediator promoting mitosis and angiogenesis in liver fibrosis, is an ideal target for evaluating liver fibrosis. VEGFR2 can be used as an effective monitoring tool for liver fibrosis monitoring, and the limitations of ultrasound elastography can be overcome by evaluating liver fibrosis through angiogenesis imaging. This study suggests that ultrasound molecular imaging is of increased value in the diagnosis of liver fibrosis and cirrhosis in rats, and another study has reached a similar conclusion. In addition, it has been found that ultrasonic molecular imaging (USMI) of CD34 targeted microbubbles is also a potential method to evaluate liver pathophysiological changes (Barr 2022). The expression of CD34, an endothelial marker with no or low expression of hepatic sinus endothelial cells in physiological states, is positively correlated with the degree of liver fibrosis. Miao and colleagues evaluated USMI’s ability to distinguish liver fibrosis, suggesting that in a rat model, USMI with CD34-targeted microbubbles can be used as a stand-alone method to assess fibrosis (Qiu et al. 2021). As a noninvasive, inexpensive and widely available technique, ultrasonic molecular imaging may become a key technique for evaluating and monitoring chronic liver disease in the future by adding multi-parameter ultrasound (Barr 2022).

#### 3.1.2. Treatment of liver fibrosis

Currently, in terms of liver fiber therapy, stem cells with the ability to regenerate and repair damaged liver cells have become a breakthrough in research. Bone marrow mesenchymal stem cells (BMSCs), a kind of stem cells derived from bone marrow, have the ability of cross-system, cross-embryonic differentiation, and high self-renewal, and have potential therapeutic potential and advantages in the cell therapy of chronic liver fibrosis. However, inadequate homing is a major challenge in therapy due to the low efficiency of targeted homing of bone marrow mesenchymal stem cells, resulting in their ability to differentiate into liver cells far below the number required for liver repair. Liver growth factor (HGF), a pleiotropic cytokine with key anti-fibrotic and anti-apoptotic effects during liver regeneration, induces bone marrow mesenchymal stem cell migration by binding to c-MET on the HGF-MET axis, which can influence the migration of bone marrow mesenchymal stem cells in vitro (Jalili et al. 2010). Therefore, some scholars have studied the combination of ultrasound-targeted microbubble destruction technology with BMSCs and HGF to help repair liver fibrosis (Sun et al. 2020), and their research results show that the stable expression of HGF in BMSCs and the application of UTMD technology contribute to the homing of BMSCs and can further improve the remission of liver fibrosis (Jiang et al. 2013; Huang et al. 2017; Yang et al. 2021). In addition, previous research by the team found that the application of UTMD can enhance the homing of bone marrow mesenchymal stem cells to the acutely injured liver, resulting in better therapeutic outcomes. Therefore, BMSCs and HGF combined with UTMD technology is expected to become a new method for the treatment of liver fibrosis (Table 5).

**Table 5.** MB Mediated diagnosis and therapy for liver fibrosis.

Applications	Microbubble	Target/mechanism	Technical characteristics	Models	References
Gene Therapy (Anti-fibrotic)	Plasmid shRNA-IGFBPrP1 + MB	Inhibition of hepatic stellate cell activation; Downregulation of TGF-β expression; Degradation of EMC	Hedgehog signaling pathway; Ultrasound for microenvironmental repair;	Rat; Liver Fibrosis	Ren et al. (2019)
Gene Therapy (Anti-fibrotic)	HGF+MB(Hepatocyte growth factor)	Promotion of apoptosis of hepatic stellate cells; Regulation of EMC synthesis; Modulation of inflammatory response; anti-inflammatory;	Drug loading; Targeted release	Rat; Liver Fibrosis	Wang et al. (2009)
Diagnosis + Gene therapy (Anti-fibrotic)	Bio-MB + Bio-CNLP + pCDH-HGF	Reduction of hydroxyproline levels; promotion of liver cell Regeneration; reduction of collagen deposition; Anti-inflammatory;	Ultrasound molecular imaging; Quantifying the extent of fibrosis	Rat; Liver Fibrosis	Zhang et al. (2013)
Gene therapy (Silencing of genes)	pMD18-T/HGF + Sonovue	Inhibition of TGF-β expression; Regulation of EMC synthesis; Improvement of liver function; stimulation of protein synthesis;	Drug loading; Targeted release; Ultrasound-mediated gene transfer	Rat; Liver Fibrosis	Jiang et al. (2013)
Gene therapy (Silencing of genes)	microRNA + MB	Inhibition of TGF-β signaling pathway; Decreased collagen synthesis;	TGF-β siRNA-loaded Microbubbles; ultrasound-targeted delivery inhibits liver fibrosis	Rat; Liver Fibrosis	Yang et al. (2013)
Medication (Anti-fibrosis)	HCPT+MBs	Inhibits hepatic stellate cell growth; Reduces collagen deposition; Modulates inflammatory response;	Drug loading (hydroxycamptothecin); Ultrasonic cavitation to enhance drug penetration; Synergistic effect of UTMD	Rat; Liver Fibrosis	Chen et al. (2023)

### 3.2. UTMD is used for diagnosis and treatment of intrahepatic focal lesions

#### 3.2.1. Intrahepatic focal lesion imaging

As a noninvasive, low-cost, and convenient imaging method, ultrasound is routinely used as the preferred monitoring method. Contrast-enhanced ultrasound is an effective and reliable tool that can be used to clarify diagnostic doubts at the end of a routine ultrasound or as a routine examination for specific patients, such as those with tumors and cirrhosis. Because the real-time imaging capability of CEUS is particularly effective for continuous monitoring of arterial phase douche, it can eliminate CT and MRI phase errors due to differences in patient cardiac output or cycle time. In CT and MRI, phase errors in the arterial phase may directly affect the detection of arterial phase high intensification (APHE), and this feature of contra-enhanced ultrasound is critical to characterizing focal nodules of unknown nature in liver fibrosis.

Compared with enhanced CT, the sensitivity and specificity of CEUS in the diagnosis of intrahepatic focal lesions were generally improved (Battaglia and Cervelli 2017). In a prospective study, 121hepatic nodules were scanned with CEUS and CT enhancement (Quaia et al. 2009). The results showed that CEUS could distinguish abnormal perfusion caused by ablation from local APHE associated with recurrence, thus helping to identify uncertain APHE on CT or MRI scan. In addition, real-time CEUS shows that most malignant tumors with low blood vessels (such as metastases) show transient APHE and then disappear rapidly. Therefore, CEUS is used to determine the characteristics of small and unknown liver lesions with low density on CT and low signal intensity on MRI, to determine whether the lesions are resectable or require chemotherapy, and to stage the disease more accurately.

The integrated accumulation of CEUS ultrasonographic findings can be used to determine the CEUS features of a variety of common liver lesions, such as complex liver cysts, hepatocellular carcinoma during screening, incidental hemangiomas, or focal fat deposition of the liver, or segmental liver lesions. Because of its portability, ultrasound can be used in intensive care, interventional and intraoperative environments, and can be used to target and evaluate immediate treatment response and/or residual lesions during ablation of hepatocellular carcinoma (Atri et al. 2022). CEUS is reported to be valuable in providing diagnostic features of focal intrahepatic lesions of an unknown nature on CT, magnetic resonance imaging (MRI) and positron emission tomography (Dietrich et al. 2020). In addition, contrast-enhanced ultrasound should be chosen as the follow-up imaging mode of uncertain nodules after all CT and MR examinations before biopsy. The main purpose of CEUS in patients with no history of liver cirrhosis is to distinguish between benign and malignant intrahepatic focal lesions. Therefore, contrast-enhanced ultrasound can be used to determine whether the detected intrahepatic focal lesions need further examination or surgery.

#### 3.2.2. Treatment of intrahepatic focal lesions

##### 3.2.2.1. Hepatocellular carcinoma (HCC)

The traditional treatment of hepatocellular carcinoma includes surgery, drug therapy, trans arterial embolization, etc. In recent years, immunotherapy has become an important means of tumor treatment due to the rapid development of tumor immunotherapy. Compared with traditional therapies that directly act on the tumor itself, tumor immunotherapy can enhance the immune defense mechanism against the tumor, stimulate the body’s immune system, indirectly attack tumor cells, and reshape the immune microenvironment. On the one hand, it can enhance immune-mediated tumor cell death by promoting the recognition and elimination of target cells carrying tumor antigens by immune tumor cells. However, due to the specific characteristics of tumor microenvironment, such as hypoxia, tumor vascular malformation and immune escape, as well as the limitations of current immunotherapy, such as off-target toxicity, insufficient drug penetration and accumulation, and immune cell dysfunction, the application of immunotherapy is limited. Ultrasound targeted microbubble destruction (UTMD) therapy helps to reduce the adverse events associated with immunotherapy and regulate the tumor immunosuppressive microenvironment by enhancing the effect of immunotherapy (Han et al. 2022). In addition, many related studies have also proved that local ultrasound irradiation can trigger the targeted release of drugs and foreign genes carried by microbubbles and promote gene transfer to deep tissues (Di Ianni et al. 2019), thus achieving higher therapeutic efficiency (Zhou et al. 2010; Guo et al. 2020). Therefore, UTMD shows a good prospect in improving the efficacy of immunotherapy.

MicroRNA (MiRNA) plays a key role in the occurrence and development of cancer (Ladeiro et al. 2008). In recent years, more and more evidence shows that miRNA also plays an important role in regulating host immune response (Diener et al. 2022). Programmed cell death-1 (PD-1) and programmed death ligand 1 (PD-L1) are important factors in maintaining the stability of tumor immune microenvironment, which can help cancer cells escape from immune surveillance in tumor microenvironment. The binding of overexpressed PD-L1 on tumor cells to PD-1 on tumor infiltrating lymphocytes (TIL) can regulate cell proliferation, growth and survival, thus inhibit immune cell proliferation. Previous monotherapy by inhibiting PD-1/PD-L1 pathway has been successful in early clinical trials, but free PD-L1 checkpoint inhibitors have strong toxicity and adverse reactions. In 2022, our team prepared nano-lipid microbubbles carrying PD-L1 antibody (Ab) and miR-424 and evaluated their synergistic immunotherapeutic activity through the mouse model of H22 hepatoma transplantation (Chen et al. 2022). This study found that in vitro and in vivo models, targeted nano-bubbles loaded with PD-L1 antibody, and miR-424 gene could enhance the sensitivity of tumor cells to drug therapy, at the same time activate T cells, block PD-L1 immune checkpoints, mediate tumor cell apoptosis and inhibit the progression of hepatocellular carcinoma. Our team also achieved satisfactory results in the treatment of liver tumors by using drug-loaded lipid nanobubbles and tumor-specific monoclonal antibodies (Tan et al. 2021).

In addition, the combination of intra-arterial therapy and UTMD technology was also included in the study. Image-guided intra-arterial (IA) therapy (hepatic arterial chemoembolization (HAIC) or trans arterial chemoembolization (TACE) is often used in the treatment of primary or secondary liver cancer. HAIC directly delivers high concentrations of chemotherapeutic drugs and locally targets to the tumor, while TACE combines locally targeted drug delivery with simultaneous tumor arterial embolization. The purpose of these two treatments is to ensure the maximum dose of chemotherapeutic drugs when the systemic toxicity is reduced to the lowest, but because of the limited delivery of drugs to the target tumor, the change of tumor microenvironment and local tumor recurrence around the region, its clinical effect is still not satisfactory. Therefore, in order to reduce the drug concentration in the blood and the side effects caused by drug miss, some researchers have prepared a new drug delivery carrier, such as human serum albumin nanoparticles (HSA-NPs) for IA therapy (Kim et al. 2021). The drug is effectively delivered to the tumor site through UTMD, which has the advantages of improving drug loading efficiency, preventing drug degradation, and producing intracellular permeation. Nanoparticles make the drug release continuously at the tumor site that promotes the selective delivery of drugs and the functional release of targets and achieves a better anti-tumor effect (Lee et al. 2019) (Table 6).

**Table 6.** MB Mediated therapy for liver cancer.

Therapeutic method	Disease type	Animal model	Microbubble	Ultrasound parameter*	Outcomes	References
Antibody Immunotherapy	Hepatocarcinoma	Mice; Liver cancer	PD-L1 mAb/DOX NBs	1.0 MHz; 45s; 50%.	PD-L1 mAb/DOX NBs had good aggregation in tumors; Blocking the PD-1/PD-L1 pathway can improve the ability of immune cells to kill tumors;DOX induced tumor cell apoptosis and immunogenic cell death	Chen et al. (2022)
Immunotherapy	Hepatocarcinoma	Mice; Liver cancer	sPD-1/Ce6-NBs	1.1 MHz; 1.8 W/cm^2^; 2 min; 50%	Nbs enhanced the accumulation of Ce6 and sPD-1 tumor targeting; sPD-1 blocked the PD-1/PD-L1 pathway. ICIs combined with STD synergistically enhance the anti-tumor immune response	Tan et al. (2021)
Transarterial chemoembolization (TACE)	Hepatocarcinoma	Rabbit; VX2 liver cancer	DOX-NPs-MB complex in Lipiodo	3.5 Hz; 15 min	Ultrasound-mediated sound hole effect enhanced the therapeutic effect; Doxorubicin-loaded microbubbles combined with TACE can monitor drug delivery to tumor tissue in real time and enhance the efficacy of TACE	Kim et al. (2021)
Drug Therapy	Human HepG2 cells	/	DR5-DLLM (Docetaxel)	0.5 W/cm^2^; 45s	The expression of Bcl-2 and NF-κB was down-regulated, and the expression of Caspase-8 and DR5 was up-regulated; Enhanced the effect of HepG2 cell cycle arrest, cell proliferation inhibition and cell apoptosis induction	Yang et al. (2021)
Gene Therapy	Hepatocarcinoma	Mice; Liver cancer	miRNA-122 + antimiR-21-PLGA-PEG-NPs	1.8 Hz;1min; 5.4 MPa	The tumor volume was significantly reduced. The proliferation, migration and invasion of HCC cells were decreased. To improve the sensitivity of hepatocellular carcinoma cells to adriamycin.	Chowdhury et al. (2018)
Microwave Ablation	Hepatocarcinoma	Rabbit; VX2 liver cancer	Prothrombin; the microbubble SonoVue	/	Ultrasound microbubbles can enhance the effect of microwave ablation of rabbit VX2 liver cancer. The necrosis and apoptosis rates of hepatocellular carcinoma cells were increased. There was no serious liver function damage	Shi et al. (2019)

Ultrasound Parameter*: Frequency (MHz); Intensity (W/cm^2^); Time (s/min); Duty cycle (%); Acoustic pressure (MPa).

##### 3.2.2.2. Acute liver injury

Severe acute liver injury (ALI) is a syndrome caused by a large number of hepatocyte damage and progressive deterioration of liver function, which will eventually develop into brain and renal failure without intervention, with a high mortality rate. Some studies have shown that stem cells can be used as a source of hepatocyte regeneration to repair damaged liver, but the localization ability of bone marrow mesenchymal stem cells (BMSCs) to the target tissue is not satisfactory, which affects the therapeutic effect. Ultrasound-targeted microbubble destruction (UTMD) has previously been reported to promote the homing of transplanted stem cells to ischemic myocardium, so there are studies to explore the promoting effect of BMSCs on liver homing in rats with UTMD for acute liver injury, and evaluate its therapeutic effect on repairing injured liver (Sun et al. 2018). They isolated BMSCs from femur and tibia of rats, and verified the appropriate ultrasound parameters with tumor necrosis factor-α (TNF-α) and stromal cell-derived factor-1 (SDF-1). After treatment of BMSC with UTMD, the number of BMSCs in UTMD group was significantly higher than that in other control groups, the apoptosis rate of hepatocytes was significantly lower than that in BMSCs group, and the pathological changes of liver tissue were significantly alleviated. This study suggests that UTMD therapy can effectively promote the liver homing of BMSCs, so UTMD therapy seems to be an effective noninvasive method to assist BMSC homing to repair damaged liver.

## 4. The safety of ultrasonic microbubbles

The currently used ultrasound contrast agents (UCAs) in clinical applications are microbubbles. When injected through peripheral veins or through body cavities, they can reach the target organs or tissues, but they cannot penetrate the vascular endothelium to enter the interstitial space. UCA is not excreted through the kidneys and can be safely used by patients with renal insufficiency. UCA does not contain iodine and there is no evidence of its effect on thyroid function; it has no ionizing radiation and can be used for infants and children. The most common adverse reactions of UCA include headache, nausea, chest pain and discomfort, as well as skin symptoms such as itching and urticaria. Severe cases may result in respiratory and cardiac arrest, anaphylactic shock and loss of consciousness (Hu et al. 2019; Strom et al. 2025). According to multiple studies, the incidence of adverse events of UCA is extremely low. It is reported that the incidence of serious adverse events or reactions of sulfur hexafluoride microbubbles is less than 0.1‰ (Walker et al. 2024); while the safety of perfluorobutane microspheres still needs further research due to limited research data. According to the post-marketing investigation results in South Korea, the incidence of adverse events of perfluorobutane microspheres is 3.23% (99/3066), and the incidence of serious adverse events is 0.03% (1/3066).

A retrospective evaluation was conducted in all patients who received CEUS with sulfur hexafluoride microbubbles (SonoVue) injected intravenously in 24 patients from January 2006 to April 2019 to evaluate the safety of ultrasound contrast agent sulfur hexafluoride microbubbles, and to compare the frequency of adverse reactions among different patient subgroups (Shang et al. 2023). After injection of sulfur hexafluoride microbubbles, a total of 157cases of adverse reactions (153cases (0.033%) were not serious; 4 cases (0.001%) were severe), and the incidence of specific adverse reactions was 0.034% (157 thumbs 463434). Among the non-severe adverse reactions, mild was found in 66 cases (0.014%), moderate in 70 cases (0.015%) and severe in 17 cases (0.004%). The frequency of AE in liver after CEUS was 0.026%. Patients with vascular disease CEUS had the highest incidence of AE (0.092%). Sulfur hexafluoride microbubbles have low frequency of adverse reactions and few severe reactions, so they are suitable for conventional contrast-enhanced ultrasound applications. One study evaluated six pregnant women who underwent SonoVue^®^ contrast-enhanced ultrasound, none of whom had adverse fetal or maternal events (Schwarze et al. 2020).

A study on the use of ultrasound contrast agents in children came to a similar conclusion (Mao et al. 2019), and although CEUS has not been clinically approved for use in obstetrics, it may be used in the future for the diagnosis of diseases in pregnant women and children. In addition, the safety of various self-made diagnostic or therapeutic microbubbles in animals has also been verified.

## 5. Limitations and challenges of ultrasonic microbubbles

CEUS cannot improve the ability to detect small liver cancer, so the detection of hepatocyte nodules by CEUS is also limited when the nodules cannot be detected by traditional gray-scale ultrasound. When the patient is fat, the lesion is deep, or the lesion is located below the diaphragm, the detection of CEUS will be limited because of the limited penetration power of ultrasound, serious signal attenuation or gas interference in gastrointestinal tract and lung. Furthermore, obese patients often have hemodynamic changes, and the residence time of microbubbles in the target tissue is shortened. Additionally, the liver of obese patients is usually located deeper, and these factors all contribute to a significant reduction in the effectiveness of UTMD. The conventional diagnostic ultrasound has a significantly reduced sensitivity in detecting liver lesions smaller than 1 cm. Although UTMD can enhance contrast, it is difficult to break through the physical resolution limit. At the same time, small liver cancer lesions often exhibit heterogeneous blood supply characteristics, resulting in uneven distribution of microbubbles and affecting the effect of UTMD. However, some nodules that are difficult to be found by gray-scale ultrasound can be found in the later stage after microbubble injection using anatomical markers or combined with CT and MRI, and rinsing with contrast media.

Although ultrasound-targeted microbubble destruction has been widely studied in the treatment of liver diseases, there are few cases in clinical trials (Escoffre et al. 2022). In 2022, Eisenbrey et al. (2021) conducted a clinical study to evaluate the safety and preliminary efficacy of ultrasound-targeted microbubble destruction combined with transarterial radioembolization (TARE) in the treatment of hepatocellular carcinoma. The results showed that before and after ultrasound-targeted microbubble destruction, there were no significant changes in patients’ body temperature, heart rate, and blood pressure, and there were no significant differences in liver function. The ultrasound tumor ablation technique enhanced the sensitivity of tumors to radiotherapy, which may help improve tumor responsiveness and patient survival. This study demonstrated that the method combining ultrasound-triggered microbubble destruction with TARE has good safety and feasibility in the treatment of hepatocellular carcinoma and can improve treatment efficacy. Another clinical study on the use of UTMD combined with chemotherapy to treat metastatic tumor and abdominal wall tumor in liver showed that UTMD seemed to have certain effects (Shang et al. 2023), but due to differences in chemotherapy drugs and patients’ basic conditions, accurate conclusions could not be drawn (Shen et al. 2020). There is no conclusive clinical evidence to prove the efficacy of UTMD in the treatment of liver metastases (Kotopoulis et al. 2013; Dimcevski et al. 2016; Wang et al. 2018; Castle et al. 2020; de Maar et al. 2021).

## 6. Discussion, conclusion and outlook

The liver is an ideal target for the treatment of many diseases because it is a major contributor to multiple metabolic pathways and serum protein production (Tran et al. 2019). Some scholars have studied the effect of ultrasonic targeted microbubble destruction (UTMD) on the permeability of normal liver tissue (Yang et al. 2012), using Evans blue and lanthanum nitrate as tracers to measure the permeability of capillaries and cell membranes, and proved that ultrasonic targeted microbubble destruction can increase the permeability of capillaries and cell membranes in normal liver tissue, without significantly increasing hepatorenal toxicity. At present, many scholars have designed nano-delivery platforms through different methods, which are of great significance in the treatment of liver diseases or the high ultrasonic detection sensitivity of liver imaging through various characteristics of response to ultrasonic irradiation.

The goal of traditional therapies is to include as many patients as possible, so early and accurate diagnosis is crucial, which will benefit patients even more (Koyama and Brenner 2017). And imaging means at the molecular scale contribute to early diagnosis and better understanding of pathological diagnosis, and researchers are committed to design and develop vectors that can specifically target pathological biomarkers. To improve the traditional direction of treatment, the use of microbubbles to wrap drugs, has been widely studied at the nanoscale. The intrinsic properties, shell, and gas core of the microbubbles are being improved with further research, and since microbubbles is inherently responsive to ultrasound, they have considerable advantages over other clinical contrast agents used for diagnostic imaging, such as PETCT and SPECT, MRI, or X-rays (Najahi-Missaoui et al. 2020). Microbubbles have an impact on the treatment and diagnosis of human diseases at the microscopic scale (Rix et al. 2020). Their functions have also evolved from simple diagnostic contrast agents to therapeutic agents of coupling targeting agents and loading drugs, and then to multi-functional microbubbles at the present stage, which have been intensively studied for their dual potential in imaging and therapy (Fournier et al. 2023).

With the development of ultrasound imaging systems, the use of microbubbles as a tool for drug or gene delivery also has great clinical potential, especially in oncology and vascular applications. The combined application of ultrasonic microbubbles and thrombolytic enzyme can lead to rapid and minimally invasive thrombolysis and target vessel recanalization, which can be used in the treatment of stroke (Liang et al. 2021). The application of UTMD in the field of oncology has achieved significant breakthroughs. Taking cervical cancer as an example, the combination of low-frequency ultrasound and microbubbles can significantly enhance the delivery efficiency of genes. Preclinical studies have shown that when UTMD is combined with sPD1 gene therapy for cervical cancer models, the tumor inhibition rate can reach over 78% (Liu et al. 2023). Moreover, this technology has also been applied to the local ablation of solid tumors such as liver cancer and endometrial cancer, reducing the need for surgical resection. In the field of cardiology, UTMD is used for the intervention of heart valve stenosis and coronary heart disease. By releasing drugs that dilate blood vessels through microbubble rupture, it can improve myocardial perfusion and reduce the risk of cardiovascular events. The combination of microbubbles with ultrasound offers a possibility to optimize drugs and drug delivery systems by improving drug pharmacokinetics and delivery to targets, and a new generation of microbubbles also opens new prospects for targeted molecular imaging. As the application and innovation of ultrasonic microbubbles flourish in multiple fields (oncology, cardiovascular disease, inflammation, etc.), the combination of ultrasonic targeted destruction technology and ultrasonic microbubbles will play a crucial role in the treatment and diagnosis of liver diseases in the future.

Ultrasound-targeted microbubble destruction technology (UTMD) is an innovative medical technology that integrates multiple disciplines. Its development requires in-depth collaboration among the academic community, industry, and regulatory authorities. The academic community mainly undertakes basic research in UTMD technology development, including optimizing microbubble materials, exploring ultrasound parameters, and studying biological effects; while the industry is responsible for technology transfer, such as equipment manufacturing, large-scale production of microbubbles, and support for clinical trials. UTMD involves multiple aspects such as microbubbles and ultrasound equipment, so in order to better ensure its safety, regulatory authorities are needed to assist in formulating unified safety standards and regulatory paths. In the future, the three parties need to further deepen their collaboration to jointly solve key issues such as large-scale production of microbubbles, standardization of treatment, and long-term safety assessment. Through establishing closer cooperation, UTMD is expected to enter clinical practice more quickly, providing new treatment options for precision medicine.

## Data Availability

The original contributions presented in this study are included in the article material. Further inquiries can be directed to the corresponding author(s).
